# Comparative Analysis of Artificial Intelligence Virtual Assistant and Large Language Models in Post-Operative Care

**DOI:** 10.3390/ejihpe14050093

**Published:** 2024-05-15

**Authors:** Sahar Borna, Cesar A. Gomez-Cabello, Sophia M. Pressman, Syed Ali Haider, Ajai Sehgal, Bradley C. Leibovich, Dave Cole, Antonio Jorge Forte

**Affiliations:** 1 Division of Plastic Surgery, Mayo Clinic, Jacksonville, FL 32224, USA; 2 Center for Digital Health, Mayo Clinic, Rochester, MN 55905, USA; 3 Department of Urology, Mayo Clinic, Rochester, MN 55905, USA

**Keywords:** artificial intelligence, natural language processing, large language model, machine learning, ChatGPT, Bard

## Abstract

In postoperative care, patient education and follow-up are pivotal for enhancing the quality of care and satisfaction. Artificial intelligence virtual assistants (AIVA) and large language models (LLMs) like Google BARD and ChatGPT-4 offer avenues for addressing patient queries using natural language processing (NLP) techniques. However, the accuracy and appropriateness of the information vary across these platforms, necessitating a comparative study to evaluate their efficacy in this domain. We conducted a study comparing AIVA (using Google Dialogflow) with ChatGPT-4 and Google BARD, assessing the accuracy, knowledge gap, and response appropriateness. AIVA demonstrated superior performance, with significantly higher accuracy (mean: 0.9) and lower knowledge gap (mean: 0.1) compared to BARD and ChatGPT-4. Additionally, AIVA’s responses received higher Likert scores for appropriateness. Our findings suggest that specialized AI tools like AIVA are more effective in delivering precise and contextually relevant information for postoperative care compared to general-purpose LLMs. While ChatGPT-4 shows promise, its performance varies, particularly in verbal interactions. This underscores the importance of tailored AI solutions in healthcare, where accuracy and clarity are paramount. Our study highlights the necessity for further research and the development of customized AI solutions to address specific medical contexts and improve patient outcomes.

## 1. Introduction

Patient education and post-operative follow-up are essential for enhancing the quality of care and patient satisfaction as they equip patients with vital information and help prevent complications. Although individual meetings with healthcare professionals are considered the benchmark in patient management, research indicates that integrating resources into commonly used devices such as smartphones can be highly effective [1,2,3]. Educational resources for patients are vital in managing care, especially after surgery. These resources must be concise, actionable, precise, and easy to understand at a sixth-grade level. Several methods can be utilized to evaluate the readability, ease of understanding, and complexity of this information such as the Flesch–Kincaid Grade Level and the Hemingway Readability Tool [4,5,6]. 

Artificial intelligence (AI)-enabled tools have emerged as valuable assets in healthcare, particularly in patient management and individualized decision-making processes [7,8]. Numerous initiatives have focused on developing systems capable of engaging in meaningful, intelligent dialogues with patients [3,9]. Chatbots have become one of the most valuable information sources for patients worldwide, especially with recent technological advancements. For instance, in India, an AI-powered chatbot assists patients by responding to their queries and facilitating appointment scheduling. This chatbot utilizes decision tree classifier and dimensionality reduction techniques to enhance its effectiveness [10]. These conversational agents serve various roles in healthcare, from collecting essential patient information to offering preliminary diagnoses. They can also predict the patients’ risk assessment for different disorders using sophisticated algorithms like logistic regression and support vector machines (SVM) [11].

By employing natural language processing (NLP) techniques, these AI-powered virtual assistants (AIVA) can comprehend conversations and respond appropriately, utilizing their training data to execute specific tasks effectively [12]. These systems are envisioned as alternatives to healthcare professionals for performing routine patient education tasks both efficiently and cost-effectively, thereby reducing the workload of healthcare workers [13,14,15]. To further assess their efficacy, particularly in answering patient queries post-surgery, we aimed to compare these AI tools with advanced large language models (LLMs) such as Google’s BARD and OpenAI’s Generative Pretrained Transformer (GPT), which represent the forefront of NLP tools. Their ability to learn from human input and engage in coherent interactions positions them as rapid and accessible resources for individuals including patients to seek information and answers to their queries [16,17,18]. 

Research indicates substantial issues concerning the accuracy of health-related information dispensed by widely used conversational AI virtual assistants including Google Assistant, Siri, and Alexa [19]. These assistants, not being tailored for medical scenarios, may occasionally offer detrimental guidance. Alarmingly, up to 29% of their responses could cause harm, and more worryingly, 16% have the potential to lead to fatal outcomes. This highlights the critical need for the cautious use of these tools for medical information and the importance of developing more personalized and specialized AI assistants in healthcare contexts [19]. That said, there is a substantial body of research focused on the application of LLMs and AIVAs in specialized patient education tasks. These studies evaluate the feasibility, accuracy, and suitability of these technologies for responding to inquiries across various medical fields [9,15,20,21,22,23]. Some studies have enhanced chatbots using LLMs, successfully simulating the patient–physician dynamic. This advancement has enabled chatbots to effectively comprehend patient inquiries and offer accurate advice [24]. LLM-based tools, while innovative, exhibit inherent limitations in delivering consistently accurate and reliable responses. Their performance is notably influenced by the volume and nature of the information provided as well as the type and quality of the prompts received [25].

Up until now, a thorough comparison between AIVA and LLMs, especially focusing on the novel feature of ChatGPT-4, which includes the capability for verbal conversations, has not been conducted. Our study compares our custom AIVA for post-surgery patient queries with leading LLMs, BARD and ChatGPT, to address this gap. We aimed to assess the performance of LLMs that are currently accessible to the public, without the use of a retrieval-augmented generation method.

## 2. Methods

Drawing from our prior research [2,15], the AIVA was trained using IBM Watson Assistant by inputting three variations of questions and one standard answer for each of ten frequently asked topics in plastic surgery, integrating the system with the Slack application for easy access. During a 3-week testing phase, the chatbot’s responses were refined and improved based on its ability to correctly interpret and answer the sample questions provided by the research team. We compiled 242 frequently asked questions by patients undergoing surgery, categorizing them into ten prevalent topics: post-surgical pain, pain medication, post-surgical nausea, dietary recommendations, warning signs, follow-up care, suture removal, surgical drains, recovery period, and scar development. AIVA was migrated to Google Dialogflow, and questions from these topics were verbally presented to AIVA over the phone by 26 adult patients recruited randomly from the plastic surgery department at Mayo Clinic Florida [2]. We evaluated AIVA’s performance in terms of accuracy, knowledge gap, and overall appropriateness. On 16 November 2023, we conducted a comparative study by submitting the same set of questions to ChatGPT (version GPT-4, OpenAI) [18] and Google BARD (the predecessor to Gemini) [26]. Each question was introduced in a standalone session to maintain independence from previous queries. We employed both text-based and verbal prompts for ChatGPT-4. A native American English speaker verbally presented the queries through the OpenAI ChatGPT-4 mobile app in one attempt without any follow-up questions, and the answers were recorded immediately. 

**Figure 1:**
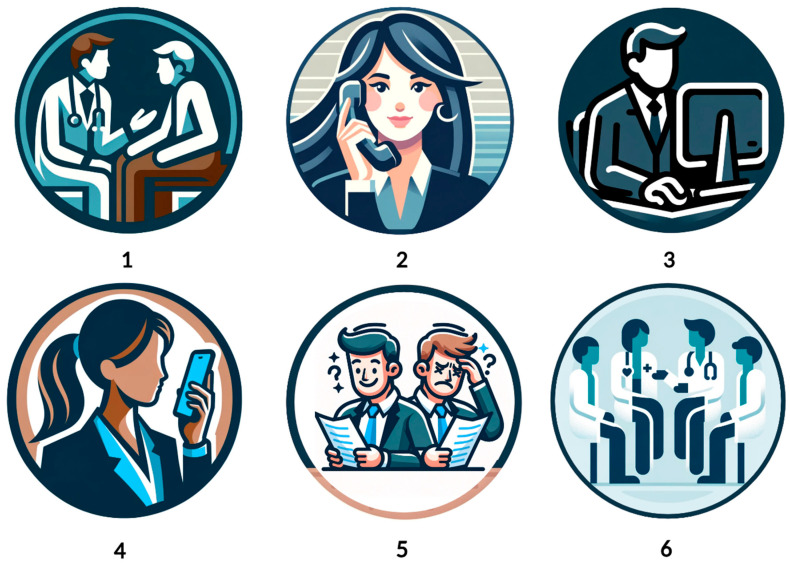
Workflow of AIVA and LLMs comparison in responding to post-operative patient queries. This process involved six steps: (**1**) Collection of 242 frequently asked post-surgical questions from patients, categorized into 10 key topics. (**2**) Development of an AIVA tailored to these questions, with patient interactions facilitated through phone calls. (**3**) Submission of the same questions to LLMs including Google BARD and ChatGPT-4 via written prompts. (**4**) Verbal querying of ChatGPT-4 with the questions and recording the responses. (**5**) Evaluation of the LLMs’ responses using specific online tools for readability and complexity. (**6**) Evaluation of response appropriateness by four healthcare professionals using a Likert scale and determination of accuracy through a binary rating system.

To assess the clarity and intricacy of the responses, we utilized the online Flesch–Kincaid [27] and Hemingway [28] readability tools. These evaluations included metrics such as the Flesch–Kincaid Grade Level and Reading Ease Score as well as the Hemingway Grade Level. Additionally, we considered word and sentence counts to assess the text complexity by measuring words per sentence, and estimated reading times in seconds using the Hemingway tool. A qualified plastic and reconstructive surgeon provided the definitive answer for each question, serving as the benchmark for our analysis. Four healthcare experts assessed the responses provided by the LLMs in comparison to the predefined correct answers. This evaluation employed a three-point Likert scale, categorizing the responses as ‘Correctly answered (3)’, ‘Correct but incomplete (2)’, or ‘Incorrectly answered (1)’. Moreover, one physician assessed the responses for accuracy, employing a binary (Yes/No) scale. We also identified knowledge gaps in each answer, categorizing them into successfully answered questions, misheard questions (for verbal prompts), questions with misinterpreted meanings, questions needing more context, and questions advising professional healthcare guidance. All data including questions and responses were meticulously recorded in an Excel sheet for comprehensive statistical analysis. One example of a provided prompt and the corresponding answers is available in Supplementary File S1. Categorical variables were summarized as the frequency (percentage) and continuous variables were reported as the median (range) and mean (standard deviation). The overall Likert score was created based on the individual rating by four raters. The results were as follows: (1) Rated incorrect by all reviewers, (2) rated incorrect or incomplete by some reviewers, and (3) rated correct by all reviewers. When we compared the evaluation for ChatGPT-4 to the same written and verbal questions, the signed rank test was used to compare the continuous or ordinal variables while McNemar’s test was used to compare the categorical variables. We also compared the evaluation for ChatGPT and AIVA to different questions on the same ten topics. Multiple questions were answered by ChatGPT and AIVA within each topic, and the mean evaluation scores for ChatGPT and AIVA were calculated within each topic and compared between the two apps using the Wilcoxon rank sum test. The comparison between BARD and ChatGPT and BARD and AIVA were conducted in the same way. All tests were two-sided with a *p* value < 0.05 considered statistically significant. The analysis was carried out using R4.2.2. (Figure 1).

## 3. Results

Our study provides a thorough comparative analysis of AIVA against two leading LLMs, BARD and ChatGPT-4, using both written and verbal prompts. 

In an evaluation of 10 topics, AIVA significantly outperformed BARD with mean accuracies of 0.9 (±0.1) for AIVA compared to 0.5 (±0.2) for BARD (*p* = 0.002). The knowledge gap was lower for AIVA (mean = 0.1) versus BARD (mean = 0.4 ± 0.2; *p* < 0.001). AIVA also had a higher Likert score, with a mean of 2.9 (±0.1) against BARD’s 1.9 (±0.4; *p* < 0.001). AIVA showed better performance than ChatGPT for written questions. AIVA had a higher mean accuracy of (0.9 ± 0.1) compared to ChatGPT (0.7 ± 0.1), with a significant *p* value of 0.014. In terms of the knowledge gap, AIVA’s mean was 0.1 against ChatGPT’s 0.2 (±0.2; *p* = 0.048). For the Likert score, AIVA again led with a mean of 2.9 (±0.1), while ChatGPT scored 2.0 (±0.3; *p* < 0.001). Regarding the evaluation of answers to verbal questions, AIVA outperformed ChatGPT. AIVA’s mean accuracy was (0.9 ± 0.1) compared to ChatGPT’s (0.5 ± 0.2), with a *p* value of 0.001. For the knowledge gap, AIVA maintained a lower mean of 0.1 versus ChatGPT’s 0.2 (±0.2; *p* = 0.028). In Likert scores, AIVA scored higher with a mean of 2.9 (±0.1) against ChatGPT’s 1.6 (±0.4; *p* < 0.001). The comprehensive results including detailed tables and plots are available in Supplementary File S2 for in-depth review.

## 4. Discussion

### 4.1. AIVA vs. Large Language Models

In the evolution of healthcare communication, we have progressed from the initial stages of employing NLP to facilitate automated spoken telephone-based dialogue systems for patient monitoring and management at home [29,30] to the development of advanced artificial intelligence-powered virtual assistants [15]. This evolution underscores the healthcare system’s ongoing quest to enhance patient communication and education.

Patients with cancer, who might not always manage regular visits to a doctor’s office, stand to gain significantly from the use of virtual assistants. These tools offer prompt access to dependable information, crucial for managing their complex treatment plans and navigating the increased risk of complications [14,31,32]. A study conducted at a Dutch radiotherapy institute involving 127 participants found a positive correlation between the ease of use and reliability of virtual assistants and their acceptance among patients. This suggests that as virtual assistants become more user-friendly and provide accurate information, they are more likely to be embraced by this patient group [14]. In our current experiment, our analysis of AIVA, ChatGPT, and BARD revealed distinctive strengths and weaknesses across three critical dimensions: accuracy, knowledge gap, and appropriateness. AIVA stood out with its higher accuracy, distinguishing itself significantly from ChatGPT and BARD, particularly in verbal interactions. This accuracy was complemented by AIVA’s narrower knowledge gap, indicating a better understanding and response accuracy to patient queries. In terms of appropriateness, as judged by Likert scores, AIVA again showed superiority, reflecting a better alignment with patient expectations and needs.

In our prior research [15], we explored the development of our AIVA system in responding to patient inquiries following surgery, where we found that it accurately addressed 92.3% of patient questions. This study also revealed that AIVA could potentially serve as a substitute for medical professionals, with participants deeming 83.3% of its responses as correct. Furthermore, evidence suggests that our AIVA offers users a high level of satisfaction and a positive experience [2]. 

When comparing various virtual assistants including Siri, Alexa, Google Assistant, and Cortana in their ability to respond to orthodontic-related patient inquiries, Google Assistant emerged as the most effective, achieving the highest mean score on a modified Likert scale [13]. However, the variance in efficacy across these platforms highlights the necessity for more specialized training in addressing patient questions within this specific domain. It is crucial to consider the origin of the information supplied by these assistants. Notably, Google Assistant predominantly sources its content from private practice websites, and these tools are generally not designed with a primary focus on patient query resolution, but rather on providing information across various topics. Moreover, the study that provided these insights had a smaller sample size compared to ours and limited its evaluation to the relevance of responses, omitting other critical dimensions of patient education that can be significant [13]. In a comparative study [33], the accuracy of ChatGPT-4, BARD, and YOU.com was evaluated in determining the stage and management of tonsillar cancer based on a patient’s clinical vignette. This study highlighted that these LLMs provided variable responses with inconsistent accuracies to identical prompts within a short timeframe, often failing to correctly determine the cancer stage, potentially impacting treatment planning. In our analysis, AIVA’s responses demonstrated significantly greater accuracy than those of ChatGPT, both in written (average 0.9 ± 0.1 vs. 0.7 ± 0.1) and verbal formats (average 0.9 ± 0.1 vs. 0.5 ± 0.2). This accuracy discrepancy was notably pronounced between AIVA and ChatGPT in verbal responses, with a *p* value of 0.001. Furthermore, AIVA received a substantially higher mean Likert score compared to ChatGPT. The mean Likert score for AIVA was nearly double that of ChatGPT for verbal prompts (2.9 ± 0.1 vs. 1.6 ± 0.4, *p* value < 0.001).

In a busy emergency room setting, Google BARD proved beneficial for assessing drug interactions [34]. Research [35] indicates that Google BARD, Cloud, and ChatGPT versions 3.5 and 4 deliver precise, coherent, and relevant responses when accessing de-identified electronic health records (EHRs). However, this efficiency hinges on data availability, which may not be consistently accessible in real-world scenarios. Notably, these language models including BARD do not autonomously search the Internet unless specifically instructed [36]. The study revealed that BARD effectively enhanced the readability of patient information, making it accessible at a sixth-grade level, in contrast to the higher reading levels (above 10) typical of materials from sources like JAMA, Cochrane, and the European Journal of Cardiovascular Nursing. Compared to ChatGPT, BARD produced more readable texts, though ChatGPT also contributed to improved readability through simplification. Notably, our study demonstrated AIVA’s superiority in terms of accuracy (*p* = 0.002), offering more precise responses than BARD, as evidenced by higher Likert scores (2.9 ± 0.1 vs. 1.9 ± 0.4, *p* < 0.001). Additionally, AIVA exhibited a significantly smaller knowledge gap than BARD across 10 assessed topics (*p* < 0.001). This trend was consistent in the comparison between AIVA and ChatGPT, with AIVA showing a markedly lower overall knowledge gap in both written and verbal forms (mean 0.1, *p* = 0.048 vs. mean 0.2 ± 0.2, *p* = 0.028). In our tests, the most common knowledge gap in ChatGPT was its frequent requests for additional context, occurring in 42 (17.4%) written queries and 33 (13.6%) verbal prompts. However, concerns about privacy are significant, as LLMs are not HIPAA compliant, leading to questions about their suitability in handling sensitive medical information. The risk of ‘hallucinations’, or the generation of incorrect or unreliable information, along with the potential for bias stemming from their training data, are notable limitations. These issues underscore the limitations of LLMs, particularly in medical contexts, where a more specialized tool like AIVA might be more appropriate.

### 4.2. Comparative Analysis of ChatGPT’s Performance

Revolutionary NLP-driven large language models like OpenAI’s ChatGPT 3.5 and 4, along with Google’s BARD, have demonstrated significant utility in various healthcare domains, encompassing medical research, patient management, and education [16,37,38]. Their ability to engage in conversational interactions with humans, coupled with rational and intelligent question-answering, marks them as invaluable tools for patient interaction and education [39]. However, in the rapidly advancing field of AI, patient safety, security, and satisfaction remain paramount considerations. 

In our study assessing ChatGPT’s responses to written and verbal prompts, we found distinct patterns in the model’s accuracy and knowledge gaps. For written responses, inaccuracies often stemmed from the misinterpretation of words or the need for additional context. Despite these gaps, successful answers displayed high accuracy, indicated by a significant *p*-value of <0.001. Notably, readability scores such as Flesch–Kincaid and Hemingway, did not correlate strongly with accuracy, though accurate responses tended to be longer and more detailed. In contrast, verbal responses by ChatGPT showed a different trend. Mishearing was a common source of error, but there was a notable correlation between the readability scores and accuracy. Accurate verbal responses often featured slightly more complex language, as reflected in higher Flesch–Kincaid Grade Levels and lower reading ease scores. Interestingly, accurate verbal responses contained slightly more words per sentence, suggesting a trend toward complex incorrect answers.

In their study, Temel et al. [40] evaluated the responses generated by ChatGPT to inquiries related to spinal cord injuries by using the most frequently searched keywords. They found that the complexity of ChatGPT’s responses, as indicated by a Flesch–Kincaid grade level of (14.84 ± 1.79), was significantly higher than that in our study, which recorded a grade level of (10.8 ± 2.2). Additionally, the readability of these responses, measured by the reading ease score, was considerably lower compared to ours (26.24 ± 13.81 vs. 42.9 ± 12.4). This disparity could stem from the intrinsic complexity of the spinal cord injury queries posed to ChatGPT, as these often encompass a wide array of detailed questions, whereas our study focused on simpler, more common inquiries. However, it is important to note that Temel et al. did not specify the exact nature of the prompts used, making a direct comparison of the inputs challenging. Their findings also highlighted that ChatGPT is not yet a substitute for professional patient education, as evidenced by its low Ensuring Quality Information for Patients (EQIP) score.

Another study [41] employed the Flesch–Kincaid and DISCERN tests to evaluate ChatGPT responses to typical orthodontics inquiries, categorizing them as general or treatment-related. This study sourced its questions from the top 41 websites, whereas our research derived questions directly from actual patient interactions. Utilizing the Flesch–Kincaid Reading Ease score and grade level, they demonstrated reading ease for treatment-related questions similar to our findings (47.67 ± 10.77 vs. 42.9 ± 12.4) but noted a higher grade level compared to ours. This echoes Tamel et al.’s findings [40]. To assess the response reliability, they applied the DISCERN tests for treatment-related questions in contrast to our approach, which involved compiling the most frequently asked patient questions. Our methodology did not differentiate questions by their nature (such as diagnosis, treatment, and overall complication management), but rather grouped them according to specific complications like scar formation, alarming signs, and nausea. We chose to evaluate the accuracy and appropriateness of responses through professional judgment and a Likert scale rating by four physicians, guided by an expert plastic surgeon’s opinion. While they reported moderate reliability in GPT responses per the DISCERN tool, our assessment yielded similar results. A study [42] demonstrated that ChatGPT effectively simplified breast cancer information to a sixth-grade reading level, enhancing both the readability and ease of comprehension. These AI-generated responses were not only more readable but also clinically appropriate, a crucial factor in patient education. The research used various readability assessment scales, noting that while the original responses were less readable than our written prompts (13 vs. 10.8 ± 2.2), the simplified responses closely matched the readability of our verbal prompts (8.9 vs. 8.8 ± 2.0). This suggests that verbal prompts yield more understandable responses than written ones, a significant benefit for patients with lower literacy levels, providing them with clearer and more concise information.

Online posts served as a resource for extracting patient queries and evaluating GPT’s readability and comprehension in answering otolaryngology-head and neck surgery-related questions [43]. Grouping 54 questions into fact, policy, and diagnosis categories, these questions yielded a reading ease score of 42.3 ± 13.1, lower than that of standard web searches. In contrast, our study found higher reading ease scores for verbal prompts and slightly higher scores for written ones. Both methods achieved similar understandability scores using the Patient Education Materials Assessment Tool (PEMAT). Further prompting improved response readability (55.6 ± 13.6), suggesting enhanced GPT performance with additional information. However, the study did not examine the impact of this on understandability as per PEMAT. Their accuracy assessment, using a 3-point scale, differed from our binary system and did not individually evaluate GPT’s knowledge gaps but considered a score of 2 as a general lack of information [43]. 

Another study compared GPT-3.5 and 4 in providing patient education on anterior cruciate ligament (ACL) injuries, assessing both the readability and information quality. Despite receiving good quality ratings, both versions struggled with readability, surpassing the average American eighth-grade level and highlighting the need for AI tools specifically designed for patient education [44]. The efficacy and safety of LLMs in real-world applications, particularly in addressing patient inquiries, is a crucial dimension [45]. For instance, a study on ophthalmology-related queries revealed significant challenges. It found that 21% of questions were misunderstood due to knowledge gaps. Nonetheless, about 60% of the responses were deemed helpful. A concerning finding was that 24.4% of the answers might range from mildly to moderately detrimental to patients. Notably, this study, like ours, did not employ the chain of thought reasoning method in prompting the LLMs. This absence could lead to subpar responses [45,46]. In contrast to our approach, this study did not benchmark the LLM responses against expert opinions, potentially impacting the accuracy of the response quality assessment.

### 4.3. Written and Verbal Prompt Responses in ChatGPT-4 Compared to BARD

**Figure 2:**
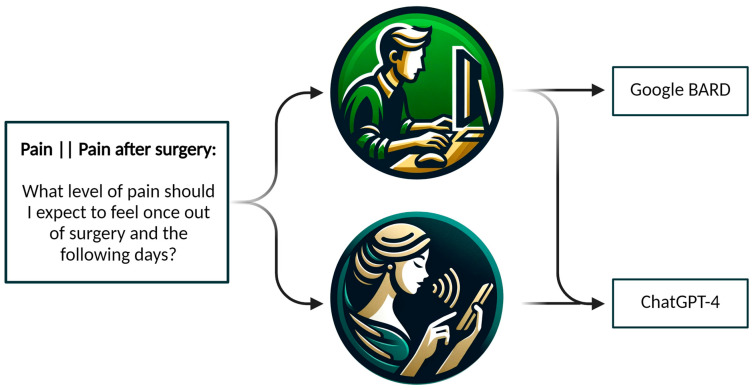
Prompting process in large language models. We introduced prompts to both BARD and OpenAI’s ChatGPT-4. Written prompts were provided to each model, while verbal prompts were exclusively given to the ChatGPT-4 mobile application by a native English speaker. The responses were then collected: written responses for the written prompts and both written and verbal responses for the verbal prompts. These responses were evaluated based on criteria such as readability, complexity, appropriateness, knowledge gap, and accuracy.

In our research, we conducted a unique investigation into ChatGPT’s capability to process verbal prompts, an area not previously explored in depth (Figure 2). 

Our comparative study involved 242 questions, delivered in both written and verbal formats, to assess their effectiveness. The results showed that written prompts often led to more accurate responses but were susceptible to misunderstandings. In contrast, verbal prompts frequently required additional context, however, there was no notable disparity in the overall knowledge conveyed between the two methods. Notably, written responses from ChatGPT demonstrated greater complexity and readability, requiring more time to process but ultimately yielding more precise answers, as evidenced by the higher average Likert scores from reviewers. This underscores the distinct variations in ChatGPT’s performance when handling verbal and written communications. When comparing ChatGPT’s written answers to those from BARD, ChatGPT consistently provided more accurate responses across the same 10 topics. However, for verbal queries, both ChatGPT and BARD showed comparable accuracy levels. The study further revealed that the knowledge gap was significantly narrower in ChatGPT-4’s written responses compared to verbal ones, whereas the gap was statistically insignificant when comparing verbal prompts between ChatGPT and BARD. Although the Flesch–Kincaid grade level indicated higher complexity for written prompts, BARD’s responses did not differ significantly in complexity from ChatGPT’s verbal prompts. Interestingly, written responses from ChatGPT were found to be easier to read than those from BARD. Verbal prompts led to more concise responses in terms of word count per sentence, but these did not exhibit a significant difference in complexity or ease of readability in comparison to BARD. Overall, the reviewers’ Likert scores remained consistent across all tools and types of prompts, indicating a uniform perception of quality regardless of the input method. When integrating LLMs into conversational interactions, particularly in healthcare settings, ethical considerations and the risk of bias present significant challenges [47]. Unlike healthcare workers, who are bound by strict ethical guidelines, interactions with AI do not currently follow these rigorous standards [47]. Research underscores the urgent need for robust dialogue safety classifiers, similar to those developed by [48], which can detect context-sensitive safety issues and mitigate biases. Moreover, there is a critical requirement to address the potential biases in responses generated by these tools stemming from the biased data used in their training. Additionally, the deployment of these tools on personal devices such as mobile phones running apps like ChatGPT-4 raises substantial privacy and safety concerns that must be diligently addressed [49]. 

### 4.4. Our Study Limitations and Future Research Directions

While our study offers valuable insights into the performance of our AI-powered virtual assistant, AIVA, BARD, and ChatGPT-4 in processing written and verbal prompts, it is important to acknowledge certain limitations that might impact the interpretation and scope of our findings.

First, our methodology did not incorporate the Ensuring Quality Information for Patients (EQIP) tool [50]. This tool could have provided a more nuanced evaluation of the information’s quality, especially in a healthcare context. Moreover, we did not utilize a range of established readability indices such as the Gunning fog index, Coleman–Liau index, automated readability index, and the SMOG index [51]. While we employed other tools for this purpose, the inclusion of these specific indices might have offered a more comprehensive understanding of the text complexity. Additionally, the “Patient Education Materials Assessment Tool (PEMAT)” [52] was not used to gauge the understandability of responses for patients. This tool could have offered valuable insights into how patient-friendly the responses were, which is particularly critical when evaluating AI-generated content in a healthcare setting.

Another limitation was the scope of our questioning. It is conceivable that not all related questions were posed, potentially omitting important aspects of AI performance. We did not use the chain of thought reasoning method [45,46] or allow for follow-up questions after the initial response to each prompt. While this approach was helpful for some comparisons, it may not fully represent real-world interactions, where iterative questioning is common and could lead to more detailed and relevant responses.

Future research should focus on key areas. Adding tools like EQIP and PEMAT would enhance the evaluation of patient information quality and provide insights into how patient-friendly AI-generated responses are. Additionally, our current tools for assessing information complexity do not fully capture the contextual use of vocabulary, its conceptual difficulty, or vocabulary complexity beyond syllables. Broadening the range of topics, engaging more patients with different backgrounds to contribute questions, and integrating the chain of thought reasoning approach would enhance the simulation of real-world interactions. Furthermore, considering a broader array of LLMs would enhance the generalizability of findings across different AI platforms. Future studies should also explore measuring empathy in AI responses, particularly in patient-centered communications, to understand its impact on patient understanding and satisfaction. Furthermore, investigating the ethical implications and policy considerations of using these tools in real-world queries is essential for ensuring the responsible and ethical deployment of AI-powered virtual assistants and LLMs across various domains [53]. Addressing these considerations will advance our understanding and optimize the performance of AIVA and LLMs in practical applications.

## 5. Conclusions

Our experiment demonstrates the effectiveness of a specialized artificially intelligent virtual assistant in providing post-operative education for patients, comparing it with large language models like OpenAI’s ChatGPT-4 and Google’s BARD. Our results show that AIVA surpassed these LLMs in delivering accurate, reliable, and contextually appropriate responses, particularly excelling in its tailored application within the healthcare domain. Specifically, our analysis of ChatGPT’s performance in processing both written and verbal prompts showed notable variations in accuracy and readability. ChatGPT performed well in written formats but had challenges with verbal prompts, requiring more context and occasionally misinterpreting information.

While ChatGPT and BARD are useful in various healthcare domains, AIVA’s superior performance emphasizes the value of customized AI solutions in healthcare settings, where the accuracy and relevance of information are critical. This suggests that while general-purpose LLMs have broad capabilities, they may not yet be optimized for specific healthcare scenarios, a niche where AIVA proves its worth.

Additionally, we acknowledge the challenges inherent in current AI technologies such as privacy concerns, potential biases, and the risk of generating unreliable information. These findings highlight the importance of the cautious and well-considered integration of AI tools in healthcare. Further experimentation is required to assess the effectiveness of a retrieval-augmented generation approach.

## Data Availability

The original contributions presented in the study are included in the article/Supplementary Material, further inquiries can be directed to the corresponding author.

## Supplementary Materials

The following supporting information can be downloaded at: https://www.mdpi.com/article/10.3390/ejihpe14050093/s1, Supplementary File S1: An example of responses from ChatGPT-4 and BARD in reaction to the written and verbal prompts; Supplementary File S2: Tables and graphs.
