# Supplementary material for: Comparative Analysis of Artificial Intelligence Virtual Assistant and Large Language Models in Post-Operative Care
Source: Eur J Investig Health Psychol Educ. 2024 May 15;14(5):1413–24. doi: 10.3390/ejihpe14050093 (PMC11119735; doi:10.3390/ejihpe14050093)
Supplement: Supplementary file 1 [file ejihpe-14-00093-s001.zip › Supp1.pdf]

Supplementary File S1. An example of responses from ChatGPT-4 and BARD in reaction to the written and verbal prompts we provided for ChatGPT, and the written prompts alone for BARD.

Type of LLM	Type of question and Provided Prompt	Received Answer
BARD	Pain || Pain after surgery<br><br>What level of pain should I expect to feel once out of surgery and the following days?	The level of pain you experience after surgery will depend on several factors, including the type of surgery you had, your individual pain tolerance, and your overall health. In general, pain after surgery is most severe in the first few days after the procedure and gradually improves over time.<br>Here is a general guideline for what to expect in terms of pain after surgery:<br>* **First 24 hours:** You will likely experience some pain, but it should be manageable with medication. You may also feel sore, swollen, and bruised at the incision site.<br>* **Days 2-3:** Your pain should start to improve, and you may be able to reduce or eliminate pain medication. However, you may still experience some discomfort, especially with movement.<br>* **Days 4-7:** Your pain should continue to improve, and you may be able to stop taking pain medication altogether. However, you may still experience some soreness or stiffness, especially with vigorous activity.<br>* **Days 7 and beyond:** The pain from your surgery should be mostly gone by this point. However, you may still experience some occasional discomfort, especially with certain activities.<br>It is important to note that this is just a general guideline. Some people may experience more pain than others, and some people may heal more quickly. If you are concerned about your pain, be sure to talk to your doctor.<br>Here are some tips for managing pain after surgery:<br>* **Take your pain medication as prescribed.** Do not take more than the recommended dosage, and do not take it more often than prescribed.<br>* **Rest as much as you need.** Getting enough rest will help your body heal and reduce pain.<br>* **Apply ice to the incision site.** This will help reduce swelling and pain.<br>* **Use a heating pad to relieve muscle soreness.**<br>* **Elevate the affected area.** This will help reduce swelling and pain.<br>* **Engage in light activity as tolerated.** This will help prevent stiffness and improve circulation.<br>* **Talk to your doctor about other pain management options.** There are a variety of non-pharmaceutical pain relief methods available, such as relaxation techniques, massage, and acupuncture.<br>If your pain is severe or does not improve with medication, be sure to contact your doctor. They may be able to adjust your pain medication or recommend other treatment options.
ChatGPT-4 (Written Prompt)		The level of pain you might experience after surgery can vary greatly depending on the type of surgery, your individual pain tolerance, the location of the surgery, and other factors such as your overall health, age, and postoperative care.<br>Types of Surgery and Expected Pain:<br>1. **Minor Surgery (e.g., dental surgery, minor lacerations)**:<br>- Mild to moderate pain<br>- Often well-controlled with over-the-counter pain relievers<br>2. **Intermediate Surgeries (e.g., hernia repair, gallbladder removal)**:<br>- Moderate pain<br>- Generally requires prescription pain medication for a few days<br>3. **Major Surgery (e.g., joint replacement, major abdominal surgery)**:<br>- Moderate to severe pain<br>- Often requires stronger pain medication and a longer duration of pain management<br>Factors Influencing Post-Surgical Pain:<br>1. **Surgical Technique**: Minimally invasive or laparoscopic procedures often result in less pain compared to open surgeries.<br>2. **Location of Surgery**: Certain areas of the body, like the abdomen or chest, may experience more pain due to the number of nerve endings and the surgery's impact on movement.<br>3. **Duration of Surgery**: Longer surgeries might result in more discomfort and prolonged recovery.<br>4. **Individual Pain Threshold**: Personal pain tolerance varies widely.<br>5. **Postoperative Care**: Adequate rest, proper wound care, and following the doctor's instructions can reduce pain.<br>6. **Pain Management**: Effective pain control strategies, including medications, physical therapy, and alternative methods like ice, heat, or acupuncture, can impact the level of pain experienced.<br>First Few Days After Surgery:<br>- **First 24-48 hours**: Often the most painful. Pain management is crucial here.<br>- **Next Few Days**: Pain typically begins to decrease. Transition from stronger pain medications to milder ones or non-pharmacologic pain management techniques might occur.<br>Tips for Managing Post-Surgical Pain:<br>1. **Follow your surgeon's advice** on medication and wound care.<br>2. **Rest**: Give your body time to heal.<br>3. **Stay ahead of the pain**: Take prescribed pain medication before the pain becomes too severe.<br>4. **Use Ice and Heat**: Depending on the type of surgery, these can reduce pain and swelling.<br>5. **Stay Active**: Gentle movements or walking as recommended by your doctor can aid in recovery.<br>6. **Report any changes**: If pain worsens or changes character, contact your surgeon.

		When to Seek Help:<br>- Pain that dramatically worsens or changes character<br>- Signs of infection (increased redness, swelling, warmth, or discharge)<br>- Fever<br>- Inability to control pain with prescribed medications<br>- New symptoms like shortness of breath, chest pain, or leg swelling<br>Every patient's experience with pain post-surgery is unique, and it's important to communicate openly with your healthcare provider about your pain levels and any concerns you have during recovery. They can provide guidance tailored to your specific situation, including adjustments in pain management if necessary.
ChatGPT-4 (Verbal Prompt)		It really depends on the type of surgery you've had. Generally, you can expect some pain after most surgical procedures, but the intensity can vary a lot. For minor surgeries, the pain may be mild and manageable with over-the-counter pain relievers. For more extensive surgeries, the pain can be significant, and your doctor might prescribe stronger pain medication. In the days following surgery, pain usually decreases gradually. Your surgical team should provide you with a pain management plan tailored to your specific situation. How are you feeling about your upcoming surgery?
