# Supplementary material for: Comparative Analysis of Artificial Intelligence Virtual Assistant and Large Language Models in Post-Operative Care
Source: Eur J Investig Health Psychol Educ. 2024 May 15;14(5):1413–24. doi: 10.3390/ejihpe14050093 (PMC11119735; doi:10.3390/ejihpe14050093)
Supplement: Supplementary file 1 [file ejihpe-14-00093-s001.zip › Supp2.pdf]

Supplementary File S2: Tables and Graphs.

Table S1. Evaluation of ChatGPT-4 answers to written and verbal questions

	Verbal (N=242)	Written (N=242)	Difference (N=242)	P value
Knowledge Gap				0.095
Successfully answered quetion	186 (76.9%)	195 (80.6%)	7 (3.8%)	
Was not able to understand	56 (23.1%)	47 (19.4%)	16 (28.6%)	
Misinterpret				0.29
No	235 (97.1%)	231 (95.5%)	6 (2.6%)	
Yes	7 (2.9%)	11 (4.5%)	2 (28.6%)	
Require.add.context				0.052
No	200 (82.6%)	209 (86.4%)	4 (2.0%)	
Yes	42 (17.4%)	33 (13.6%)	13 (31.0%)	
Redirected				1.00
No	240 (99.2%)	239 (98.8%)	3 (1.2%)	
Yes	2 (0.8%)	3 (1.2%)	2 (100.0%)	
Flesch-Kincaid Grade Level:				**<0.001**
Median (Range)	8.8 (2.1, 18.1)	10.9 (4.9, 18.3)	2.0 (-5.4, 10.3)	
Mean (SD)	8.8 (2.0)	10.8 (2.2)	2.0 (2.6)	
Flesch-Kincaid Reading Ease Score				**<0.001**
Median (Range)	57.8 (27.5, 96.2)	41.0 (9.3, 77.9)	-13.4 (-53.5, 31.4)	
Mean (SD)	57.1 (12.0)	42.9 (12.4)	-14.3 (14.4)	
Flesch-Kincaid Reading Level (Numerical)				**<0.001**
Median (Range)	5.0 (1.0, 7.0)	6.0 (3.0, 7.0)	1.0 (-2.0, 5.0)	
Mean (SD)	4.8 (1.1)	5.7 (0.8)	1.0 (1.2)	
Hemingway Grade level (#)				**<0.001**
Median (Range)	8.0 (1.0, 17.0)	11.0 (5.0, 17.0)	3.0 (-5.0, 16.0)	
Mean (SD)	8.3 (2.2)	11.7 (3.0)	3.4 (3.3)	
Hemingway # Words counts				**<0.001**
Median (Range)	66.5 (15.0, 487.0)	289.0 (45.0, 577.0)	219.0 (-434.0, 475.0)	
Mean (SD)	72.9 (41.4)	280.7 (120.5)	207.8 (114.4)	

	Verbal (N=242)	Written (N=242)	Difference (N=242)	P value
Hemingway # Sentences counts				**<0.001**
Median (Range)	5.0 (1.0, 17.0)	18.0 (2.0, 61.0)	12.0 (-3.0, 54.0)	
Mean (SD)	5.0 (2.1)	18.1 (11.0)	13.1 (10.7)	
word.per.sentence				**<0.001**
Median (Range)	14.0 (7.5, 97.4)	16.3 (6.5, 89.3)	2.5 (-89.8, 78.2)	
Mean (SD)	14.7 (6.1)	18.1 (7.7)	3.5 (10.2)	
Hemingway Reading Time (S)				**<0.001**
Median (Range)	16.0 (3.0, 61.0)	68.5 (10.0, 138.0)	52.0 (-5.0, 114.0)	
Mean (SD)	16.8 (7.7)	67.0 (29.0)	50.2 (25.9)	
Accuracy				**<0.001**
Accuracy=No	120 (49.6%)	74 (30.6%)	52 (43.3%)	
Accuracy=Yes	122 (50.4%)	168 (69.4%)	6 (4.9%)	
Avg. Likert.scale.by4				**<0.001**
Median (Range)	1.2 (1.0, 3.0)	2.0 (1.0, 3.0)	0.1 (-1.2, 2.0)	
Mean (SD)	1.7 (0.7)	2.0 (0.8)	0.4 (0.6)	
overall.Likert				**<0.001**
1=Incorrect by all reviewers	103 (42.6%)	61 (25.2%)	47 (45.6%)	
2=Not correct/completely correct by some reviewers	108 (44.6%)	124 (51.2%)	28 (25.9%)	
3=Correct by all reviewers	31 (12.8%)	57 (23.6%)	5 (16.1%)	

Table S2A. Accuracy vs. knowledge gap and complexity in ChatGPT-4 answers to verbal questions

	Accuracy=No (N=120)	Accuracy=Yes (N=122)	Total (N=242)	P value
Knowledge Gap Queries				**<0.001**
0	64 (53.3%)	122 (100.0%)	186 (76.9%)	
1	5 (4.2%)	0 (0.0%)	5 (2.1%)	
2	7 (5.8%)	0 (0.0%)	7 (2.9%)	
3	42 (35.0%)	0 (0.0%)	42 (17.4%)	
4	2 (1.7%)	0 (0.0%)	2 (0.8%)	
Flesch-Kincaid Grade Level:				**<0.001**
Median (Range)	8.0 (2.1, 18.1)	9.4 (5.4, 13.2)	8.8 (2.1, 18.1)	

	Accuracy=No (N=120)	Accuracy=Yes (N=122)	Total (N=242)	P value
Mean (SD)	8.3 (2.2)	9.4 (1.6)	8.8 (2.0)	
Flesch-Kincaid Reading Ease Score				**<0.001**
Median (Range)	61.1 (27.5, 96.2)	54.0 (30.5, 80.6)	57.8 (27.5, 96.2)	
Mean (SD)	60.5 (12.7)	53.8 (10.2)	57.1 (12.0)	
Flesch-Kincaid Reading Level (Numerical)				**<0.001**
Median (Range)	4.0 (1.0, 7.0)	5.0 (2.0, 6.0)	5.0 (1.0, 7.0)	
Mean (SD)	4.5 (1.2)	5.0 (0.9)	4.8 (1.1)	
Hemingway Grade level (#)				**<0.001**
Median (Range)	8.0 (1.0, 17.0)	9.0 (4.0, 15.0)	8.0 (1.0, 17.0)	
Mean (SD)	7.7 (2.4)	9.0 (1.8)	8.3 (2.2)	
Hemingway # Words counts				**<0.001**
Median (Range)	58.5 (15.0, 487.0)	75.0 (29.0, 200.0)	66.5 (15.0, 487.0)	
Mean (SD)	69.7 (53.0)	76.1 (25.2)	72.9 (41.4)	
Hemingway # Sentences counts				**<0.001**
Median (Range)	4.0 (1.0, 17.0)	5.0 (3.0, 12.0)	5.0 (1.0, 17.0)	
Mean (SD)	4.8 (2.5)	5.2 (1.6)	5.0 (2.1)	
word.per.sent				**0.021**
Median (Range)	13.5 (7.5, 97.4)	14.6 (9.7, 24.2)	14.0 (7.5, 97.4)	
Mean (SD)	14.6 (8.3)	14.7 (2.5)	14.7 (6.1)	
Hemingway Reading Time (S)				**<0.001**
Median (Range)	14.0 (3.0, 61.0)	18.0 (6.0, 48.0)	16.0 (3.0, 61.0)	
Mean (SD)	15.7 (8.9)	18.0 (6.1)	16.8 (7.7)	

Table S2B. Accuracy vs. knowledge gap and complexity in ChatGPT-4 answers to written questions

	Accuracy=No (N=74)	Accuracy=Yes (N=168)	Total (N=242)	P value
Knowledge Gap Queries				**<0.001**
0	27 (36.5%)	168 (100.0%)	195 (80.6%)	
2	11 (14.9%)	0 (0.0%)	11 (4.5%)	
3	33 (44.6%)	0 (0.0%)	33 (13.6%)	
4	3 (4.1%)	0 (0.0%)	3 (1.2%)	

	Accuracy=No (N=74)	Accuracy=Yes (N=168)	Total (N=242)	P value
Flesch-Kincaid Grade Level:				0.82
Median (Range)	10.4 (4.9, 18.3)	11.0 (5.3, 16.6)	10.9 (4.9, 18.3)	
Mean (SD)	10.8 (2.7)	10.8 (1.9)	10.8 (2.2)	
Flesch-Kincaid Reading Ease Score				**0.015**
Median (Range)	43.6 (19.3, 77.9)	40.2 (9.3, 71.7)	41.0 (9.3, 77.9)	
Mean (SD)	45.8 (13.0)	41.5 (12.0)	42.9 (12.4)	
Flesch-Kincaid Reading Level (Numerical)				0.13
Median (Range)	6.0 (3.0, 7.0)	6.0 (3.0, 7.0)	6.0 (3.0, 7.0)	
Mean (SD)	5.5 (0.9)	5.8 (0.7)	5.7 (0.8)	
Hemingway Grade level (#)				0.98
Median (Range)	12.0 (5.0, 17.0)	11.0 (6.0, 17.0)	11.0 (5.0, 17.0)	
Mean (SD)	11.6 (3.3)	11.8 (2.9)	11.7 (3.0)	
Hemingway # Words counts				**<0.001**
Median (Range)	187.0 (45.0, 513.0)	302.5 (108.0, 577.0)	289.0 (45.0, 577.0)	
Mean (SD)	215.0 (145.0)	309.7 (94.8)	280.7 (120.5)	
Hemingway # Sentences counts				**<0.001**
Median (Range)	8.0 (2.0, 45.0)	19.0 (3.0, 61.0)	18.0 (2.0, 61.0)	
Mean (SD)	13.1 (11.3)	20.3 (10.1)	18.1 (11.0)	
word.per.sent				**0.040**
Median (Range)	18.3 (7.6, 40.7)	15.7 (6.5, 89.3)	16.3 (6.5, 89.3)	
Mean (SD)	19.0 (6.3)	17.7 (8.2)	18.1 (7.7)	
Hemingway Reading Time (S)				**<0.001**
Median (Range)	43.5 (10.0, 123.0)	72.5 (26.0, 138.0)	68.5 (10.0, 138.0)	
Mean (SD)	51.0 (34.7)	74.1 (22.7)	67.0 (29.0)	

Table S3A. Overall Likert scale vs. knowledge gap and complexity in ChatGPT-4 answers to verbal questions

	1=Incorrect by all reviewers (N=103)	2=Not correct/completely correct by some reviewers (N=108)	3=Correct by all reviewers (N=31)	Total (N=242)	P value
Knowledge Gap Queries					**<0.001**

	1=Incorrect by all reviewers (N=103)	2=Not correct/completely correct by some reviewers (N=108)	3=Correct by all reviewers (N=31)	Total (N=242)	P value
0	48 (46.6%)	107 (99.1%)	31 (100.0%)	186 (76.9%)	
1	5 (4.9%)	0 (0.0%)	0 (0.0%)	5 (2.1%)	
2	7 (6.8%)	0 (0.0%)	0 (0.0%)	7 (2.9%)	
3	42 (40.8%)	0 (0.0%)	0 (0.0%)	42 (17.4%)	
4	1 (1.0%)	1 (0.9%)	0 (0.0%)	2 (0.8%)	
Flesch-Kincaid Grade Level:					**<0.001**
Median (Range)	7.8 (2.1, 18.1)	9.4 (5.4, 14.0)	10.0 (7.2, 12.8)	8.8 (2.1, 18.1)	
Mean (SD)	8.1 (2.1)	9.3 (1.7)	9.8 (1.7)	8.8 (2.0)	
Flesch-Kincaid Reading Ease Score					**<0.001**
Median (Range)	61.8 (27.5, 96.2)	54.3 (28.8, 77.0)	55.9 (37.1, 72.2)	57.8 (27.5, 96.2)	
Mean (SD)	61.5 (12.6)	54.1 (10.6)	53.4 (9.9)	57.1 (12.0)	
Flesch-Kincaid Reading Level (Numerical)					**<0.001**
Median (Range)	4.0 (1.0, 7.0)	5.0 (3.0, 7.0)	5.0 (3.0, 6.0)	5.0 (1.0, 7.0)	
Mean (SD)	4.4 (1.2)	5.0 (1.0)	5.1 (0.9)	4.8 (1.1)	
Hemingway Grade level (#)					**<0.001**
Median (Range)	7.0 (1.0, 17.0)	9.0 (4.0, 14.0)	10.0 (7.0, 15.0)	8.0 (1.0, 17.0)	
Mean (SD)	7.4 (2.3)	8.8 (1.8)	9.7 (2.0)	8.3 (2.2)	
Hemingway # Words counts					**<0.001**
Median (Range)	53.0 (15.0, 487.0)	72.5 (29.0, 200.0)	86.0 (42.0, 149.0)	66.5 (15.0, 487.0)	
Mean (SD)	67.2 (56.3)	74.8 (23.9)	85.2 (26.1)	72.9 (41.4)	
Hemingway # Sentences counts					**<0.001**
Median (Range)	4.0 (1.0, 17.0)	5.0 (3.0, 12.0)	5.0 (3.0, 11.0)	5.0 (1.0, 17.0)	
Mean (SD)	4.7 (2.7)	5.2 (1.5)	5.4 (1.8)	5.0 (2.1)	
word.per.sent					**<0.001**
Median (Range)	13.2 (7.5, 97.4)	14.2 (9.7, 21.2)	15.8 (11.8, 24.2)	14.0 (7.5, 97.4)	
Mean (SD)	14.5 (8.9)	14.4 (2.5)	16.0 (2.6)	14.7 (6.1)	

	1=Incorrect by all reviewers (N=103)	2=Not correct/completely correct by some reviewers (N=108)	3=Correct by all reviewers (N=31)	Total (N=242)	P value
Hemingway Reading Time (S)					**<0.001**
Median (Range)	12.0 (3.0, 61.0)	17.0 (6.0, 48.0)	20.0 (10.0, 35.0)	16.0 (3.0, 61.0)	
Mean (SD)	15.0 (9.2)	17.7 (5.9)	20.0 (6.2)	16.8 (7.7)	

Table S3B. Overall Likert scale vs. knowledge gap and complexity in ChatGPT-4 answers to written questions

	1=Incorrect by all reviewers (N=61)	2=Not correct/completely correct by some reviewers (N=124)	3=Correct by all reviewers (N=57)	Total (N=242)	P value
Knowledge Gap Queries					**<0.001**
0	15 (24.6%)	123 (99.2%)	57 (100.0%)	195 (80.6%)	
2	11 (18.0%)	0 (0.0%)	0 (0.0%)	11 (4.5%)	
3	32 (52.5%)	1 (0.8%)	0 (0.0%)	33 (13.6%)	
4	3 (4.9%)	0 (0.0%)	0 (0.0%)	3 (1.2%)	
Flesch-Kincaid Grade Level:					0.68
Median (Range)	10.7 (4.9, 18.3)	10.9 (6.8, 16.6)	11.0 (5.3, 14.7)	10.9 (4.9, 18.3)	
Mean (SD)	10.9 (2.9)	10.7 (1.9)	10.9 (1.8)	10.8 (2.2)	
Flesch-Kincaid Reading Ease Score					**0.025**
Median (Range)	43.4 (19.3, 77.9)	41.1 (17.0, 67.8)	38.3 (9.3, 71.7)	41.0 (9.3, 77.9)	
Mean (SD)	46.1 (13.6)	42.6 (11.6)	39.8 (12.2)	42.9 (12.4)	
Flesch-Kincaid Reading Level (Numerical)					0.056
Median (Range)	6.0 (3.0, 7.0)	6.0 (4.0, 7.0)	6.0 (3.0, 7.0)	6.0 (3.0, 7.0)	
Mean (SD)	5.5 (1.0)	5.7 (0.7)	5.9 (0.7)	5.7 (0.8)	
Hemingway Grade level (#)					0.31
Median (Range)	11.0 (5.0, 17.0)	11.0 (6.0, 17.0)	12.0 (6.0, 17.0)	11.0 (5.0, 17.0)	
Mean (SD)	11.1 (3.3)	11.8 (3.0)	12.0 (2.6)	11.7 (3.0)	

	1=Incorrect by all reviewers (N=61)	2=Not correct/completely correct by some reviewers (N=124)	3=Correct by all reviewers (N=57)	Total (N=242)	P value
Hemingway # Words counts					**<0.001**
Median (Range)	132.0 (45.0, 513.0)	289.0 (99.0, 577.0)	332.0 (172.0, 506.0)	289.0 (45.0, 577.0)	
Mean (SD)	195.3 (144.9)	294.2 (97.0)	342.7 (84.1)	280.7 (120.5)	
Hemingway # Sentences counts					**<0.001**
Median (Range)	7.0 (2.0, 45.0)	18.0 (3.0, 54.0)	21.0 (9.0, 61.0)	18.0 (2.0, 61.0)	
Mean (SD)	12.8 (12.1)	18.8 (9.7)	22.3 (10.4)	18.1 (11.0)	
word.per.sent					0.62
Median (Range)	17.5 (7.6, 32.5)	16.1 (7.4, 89.3)	15.8 (6.5, 26.9)	16.3 (6.5, 89.3)	
Mean (SD)	18.1 (5.6)	18.6 (9.3)	17.0 (5.3)	18.1 (7.7)	
Hemingway Reading Time (S)					**<0.001**
Median (Range)	37.0 (10.0, 123.0)	68.5 (11.0, 138.0)	79.0 (41.0, 121.0)	68.5 (10.0, 138.0)	
Mean (SD)	47.2 (34.5)	69.9 (23.8)	82.1 (20.2)	67.0 (29.0)	

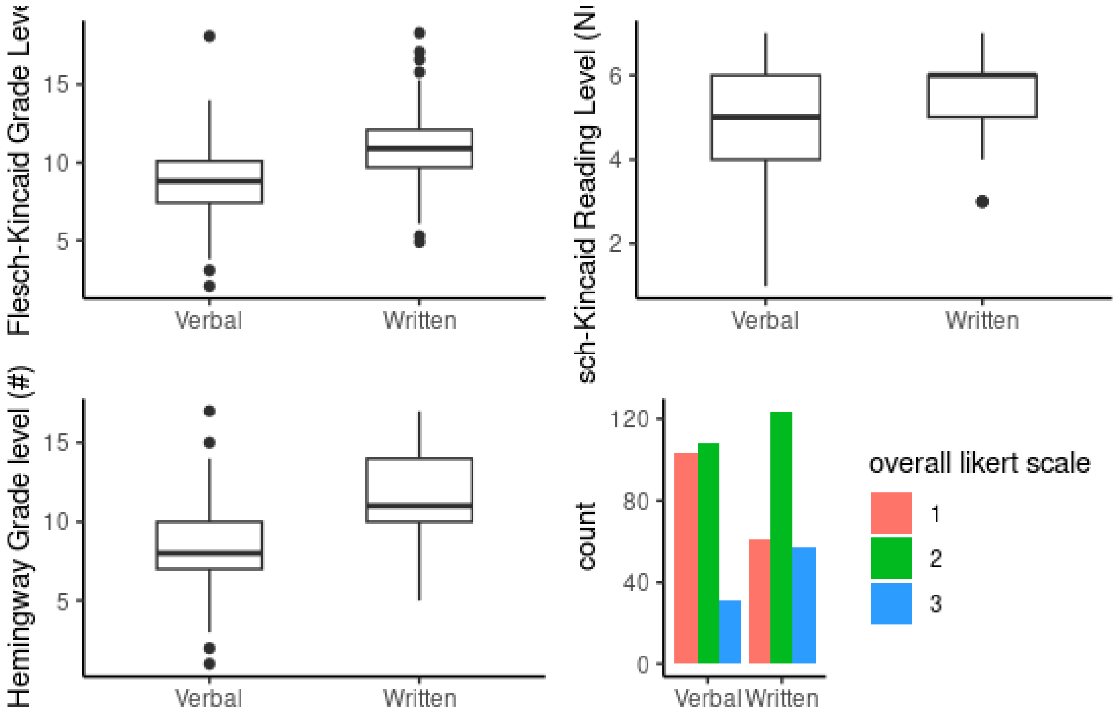

Figure S1. Evaluation of ChatGPT-4 answers to written and verbal questions

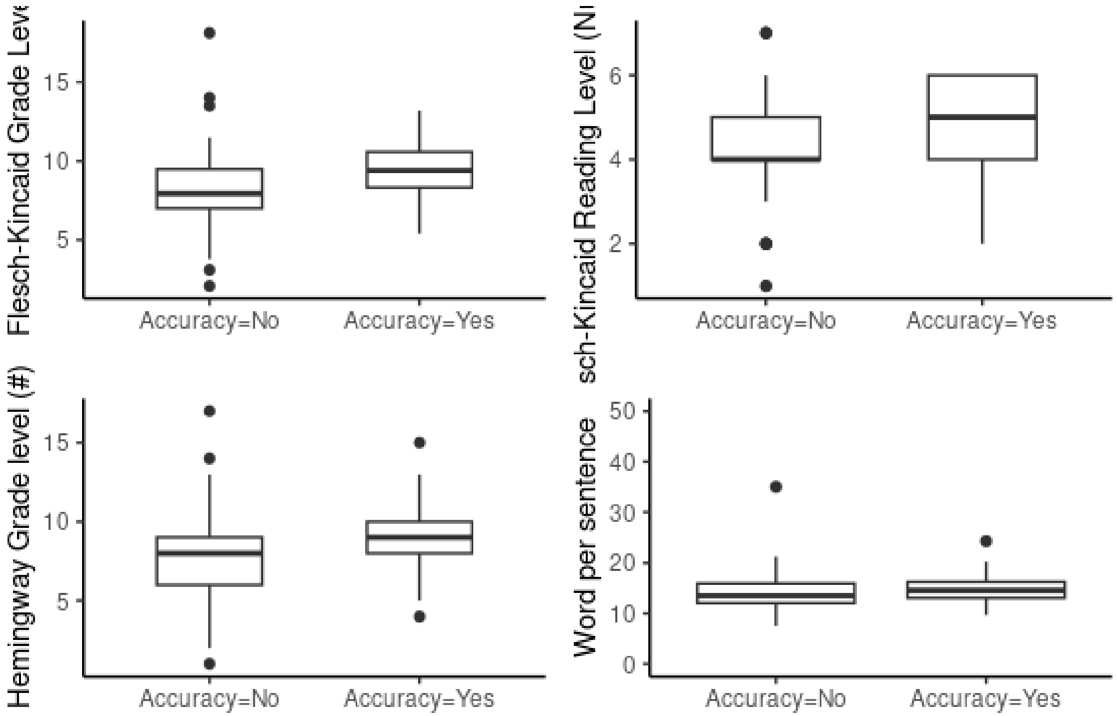

Figure S2A. Accuracy vs. complexity in ChatGPT-4 answers to verbal questions (extreme values may not show in the plots)

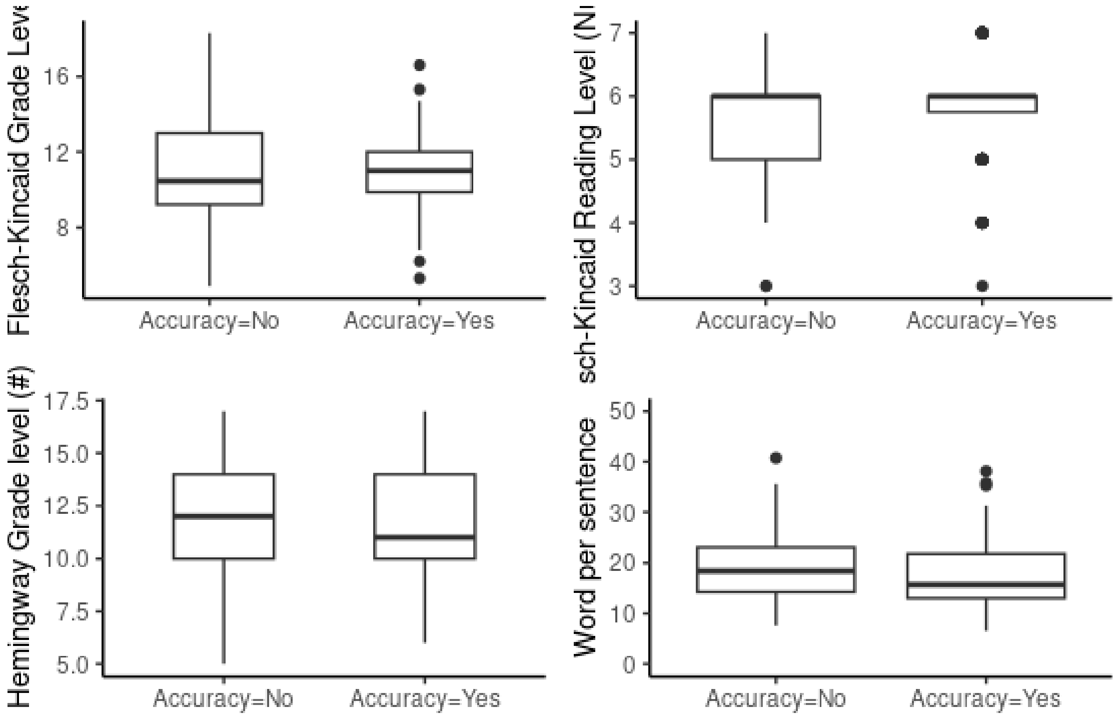

Figure S2B. Accuracy vs. complexity in ChatGPT-4 answers to written questions (extreme values may not show in the plots)

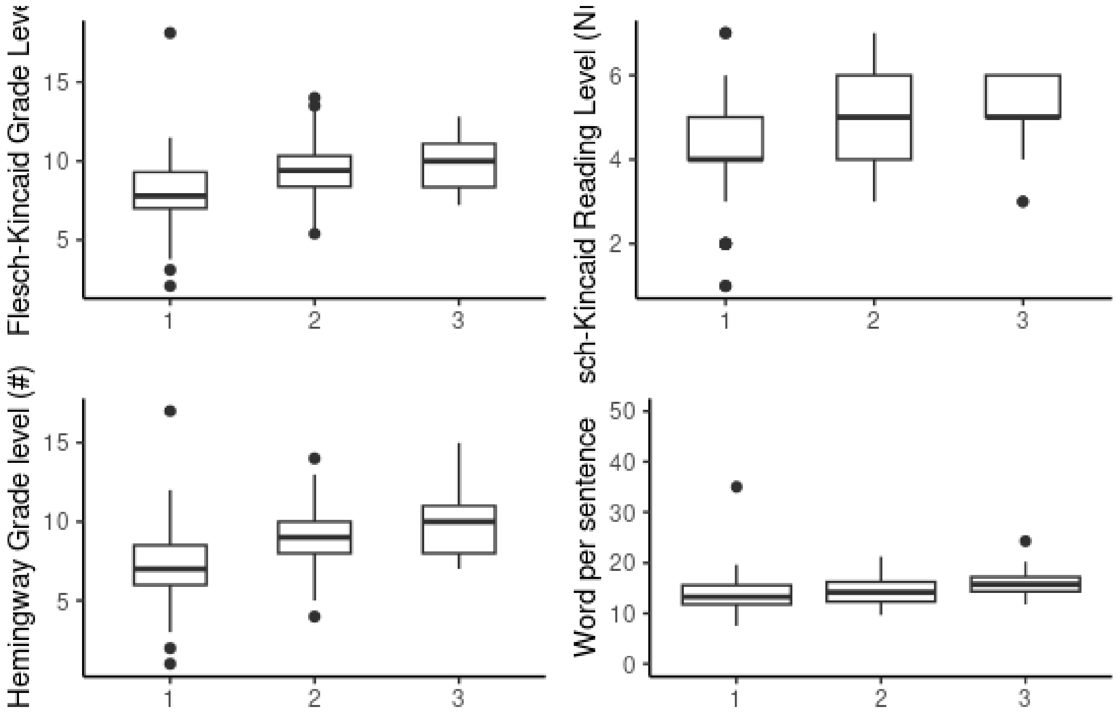

Figure S3A. Overall Likert scale vs. complexity in ChatGPT answers to verbal questions (extreme values may not show in the plots)

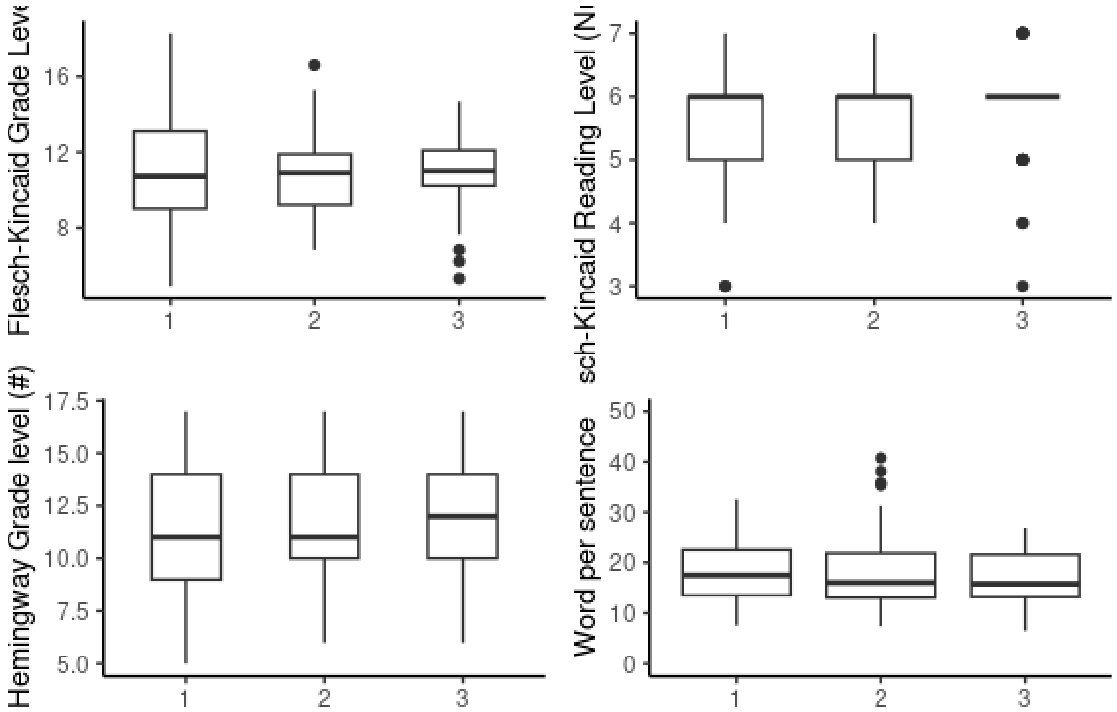

Figure S3B. Overall Likert scale vs. complexity in ChatGPT-4 answers to written questions (extreme values may not show in the plots)

Table S4A. Evaluation of AIVA answers and ChatGPT-4 answers to written questions based on the average score of same 10 topics

	AIVA (N=10)	chatGPT written (N=10)	Total (N=20)	P value
mean.accuracy				**0.014**
Median (Range)	0.9 (0.6, 1.0)	0.7 (0.5, 0.9)	0.8 (0.5, 1.0)	
Mean (SD)	0.9 (0.1)	0.7 (0.1)	0.8 (0.1)	
mean.knowledge.gap				**0.048**
Median (Range)	0.1 (0.0, 0.1)	0.1 (0.0, 0.5)	0.1 (0.0, 0.5)	
Mean (SD)	0.1 (0.0)	0.2 (0.2)	0.1 (0.1)	
mean.likert				**<0.001**
Median (Range)	3.0 (2.7, 3.0)	2.1 (1.5, 2.5)	2.6 (1.5, 3.0)	
Mean (SD)	2.9 (0.1)	2.0 (0.3)	2.5 (0.5)	

Table S4B. Evaluation of AIVA answers and ChatGPT-4 answers to verbal questions based on the average score of same 10 topics

	AIVA (N=10)	chatGPT verbal (N=10)	Total (N=20)	P value
mean.accuracy				**0.001**
Median (Range)	0.9 (0.6, 1.0)	0.6 (0.2, 0.8)	0.7 (0.2, 1.0)	
Mean (SD)	0.9 (0.1)	0.5 (0.2)	0.7 (0.2)	
mean.knowledge.gap				**0.028**
Median (Range)	0.1 (0.0, 0.1)	0.2 (0.0, 0.6)	0.1 (0.0, 0.6)	
Mean (SD)	0.1 (0.0)	0.2 (0.2)	0.1 (0.2)	
mean.likert				**<0.001**
Median (Range)	3.0 (2.7, 3.0)	1.6 (1.2, 2.4)	2.6 (1.2, 3.0)	
Mean (SD)	2.9 (0.1)	1.6 (0.4)	2.3 (0.7)	

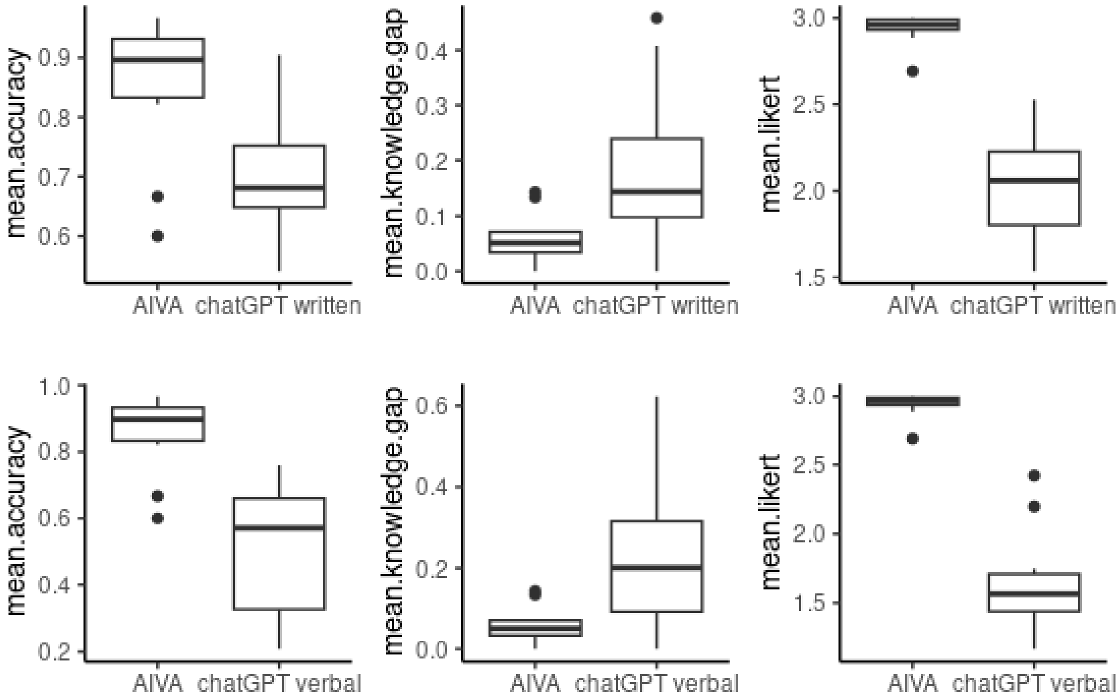

Figure S4. Evaluation of AIVA answers and ChatGPT-4 answers

Table S5A. Evaluation of BARD answers and ChatGPT-4 answers to written questions based on the average score of same 10 topics

	BARD (N=10)	chatGPT written (N=10)	Total (N=20)	P value
mean.accuracy				0.21
Median (Range)	0.6 (0.2, 0.9)	0.7 (0.5, 0.9)	0.7 (0.2, 0.9)	
Mean (SD)	0.5 (0.2)	0.7 (0.1)	0.6 (0.2)	
mean.knowledge.gap				**0.015**
Median (Range)	0.4 (0.0, 0.7)	0.1 (0.0, 0.5)	0.3 (0.0, 0.7)	
Mean (SD)	0.4 (0.2)	0.2 (0.2)	0.3 (0.2)	
mean.likert				0.50
Median (Range)	1.9 (1.3, 2.5)	2.1 (1.5, 2.5)	1.9 (1.3, 2.5)	
Mean (SD)	1.9 (0.4)	2.0 (0.3)	2.0 (0.3)	
mean.FK.grade.level				**0.003**
Median (Range)	8.9 (7.1, 9.6)	11.0 (8.7, 13.8)	9.3 (7.1, 13.8)	
Mean (SD)	8.5 (1.0)	11.0 (1.6)	9.8 (1.8)	
mean.FK.reading.score				**0.007**
Median (Range)	55.7 (51.5, 70.0)	40.6 (29.8, 57.8)	52.7 (29.8, 70.0)	
Mean (SD)	57.9 (6.7)	42.3 (9.8)	50.1 (11.4)	
mean.FK.reading.level				**0.008**
Median (Range)	4.9 (3.5, 5.4)	5.9 (4.7, 6.3)	5.2 (3.5, 6.3)	
Mean (SD)	4.7 (0.6)	5.7 (0.6)	5.2 (0.8)	
mean.HW.reading.level				**0.007**
Median (Range)	9.4 (7.7, 12.1)	14.4 (8.8, 38.9)	11.0 (7.7, 38.9)	
Mean (SD)	9.6 (1.5)	16.9 (8.8)	13.2 (7.2)	
mean.word.per.sentence				0.88
Median (Range)	17.3 (12.8, 22.2)	17.2 (11.8, 24.2)	17.3 (11.8, 24.2)	
Mean (SD)	17.9 (2.9)	17.9 (4.1)	17.9 (3.5)	

Table S5B. Evaluation of BARD answers and ChatGPT-4 answers to verbal questions based on the average score of same 10 topics

	BARD (N=10)	chatGPT verbal (N=10)	Total (N=20)	P value
mean.accuracy				0.65
Median (Range)	0.6 (0.2, 0.9)	0.6 (0.2, 0.8)	0.6 (0.2, 0.9)	
Mean (SD)	0.5 (0.2)	0.5 (0.2)	0.5 (0.2)	
mean.knowledge.gap				0.069
Median (Range)	0.4 (0.0, 0.7)	0.2 (0.0, 0.6)	0.3 (0.0, 0.7)	
Mean (SD)	0.4 (0.2)	0.2 (0.2)	0.3 (0.2)	
mean.likert				0.059
Median (Range)	1.9 (1.3, 2.5)	1.6 (1.2, 2.4)	1.6 (1.2, 2.5)	
Mean (SD)	1.9 (0.4)	1.6 (0.4)	1.8 (0.4)	
mean.FK.grade.level				0.65
Median (Range)	8.9 (7.1, 9.6)	8.7 (8.2, 11.5)	8.8 (7.1, 11.5)	
Mean (SD)	8.5 (1.0)	9.0 (1.0)	8.8 (1.0)	
mean.FK.reading.score				0.76
Median (Range)	55.7 (51.5, 70.0)	57.4 (37.7, 60.9)	57.4 (37.7, 70.0)	
Mean (SD)	57.9 (6.7)	55.5 (6.7)	56.7 (6.6)	
mean.FK.reading.level				0.79
Median (Range)	4.9 (3.5, 5.4)	4.8 (4.4, 6.3)	4.8 (3.5, 6.3)	
Mean (SD)	4.7 (0.6)	4.9 (0.6)	4.8 (0.6)	
mean.HW.reading.level				0.23
Median (Range)	9.4 (7.7, 12.1)	8.3 (7.4, 11.6)	8.6 (7.4, 12.1)	
Mean (SD)	9.6 (1.5)	8.9 (1.4)	9.2 (1.4)	
mean.word.per.sentence				**0.004**
Median (Range)	17.3 (12.8, 22.2)	14.2 (13.0, 16.8)	15.7 (12.8, 22.2)	
Mean (SD)	17.9 (2.9)	14.6 (1.2)	16.2 (2.8)	

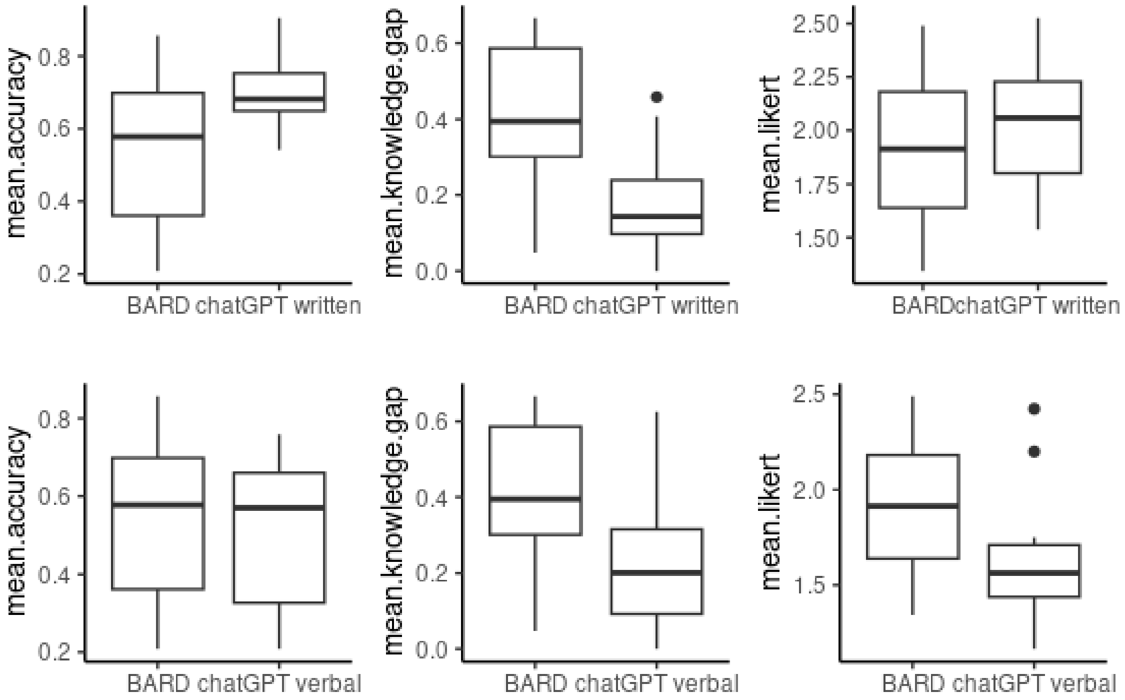

Figure S5. Evaluation of BARD answers and ChatGPT-4 answers

Table S6. Evaluation of AIVA answers and BARD answers based on the average score of same 10 topics

	AIVA (N=10)	BARD (N=10)	Total (N=20)	P value
mean.accuracy				**0.002**
Median (Range)	0.9 (0.6, 1.0)	0.6 (0.2, 0.9)	0.8 (0.2, 1.0)	
Mean (SD)	0.9 (0.1)	0.5 (0.2)	0.7 (0.2)	
mean.knowledge.gap				**<0.001**
Median (Range)	0.1 (0.0, 0.1)	0.4 (0.0, 0.7)	0.1 (0.0, 0.7)	
Mean (SD)	0.1 (0.0)	0.4 (0.2)	0.2 (0.2)	
mean.likert				**<0.001**
Median (Range)	3.0 (2.7, 3.0)	1.9 (1.3, 2.5)	2.6 (1.3, 3.0)	
Mean (SD)	2.9 (0.1)	1.9 (0.4)	2.4 (0.6)	

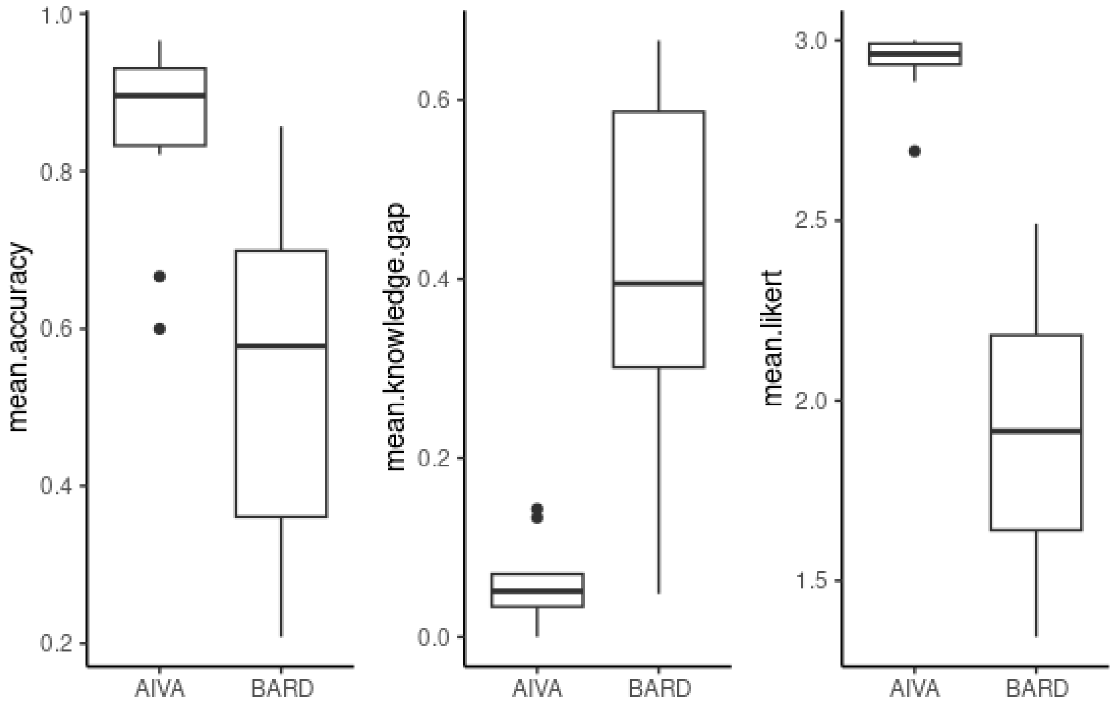

Figure S6. Evaluation of AIVA and BARD answers
